# A transcriptomic time-series reveals differing trajectories during pre-floral development in the apex and leaf in winter and spring varieties of *Brassica napus*

**DOI:** 10.1038/s41598-024-53526-x

**Published:** 2024-02-12

**Authors:** D. Marc Jones, Jo Hepworth, Rachel Wells, Nick Pullen, Martin Trick, Richard J. Morris

**Affiliations:** 1 Crop Genetics, John Innes Centre, Norwich Research Park, Norwich, NR4 7UH UK; 2 Computational and Systems Biology, John Innes Centre, Norwich Research Park, Norwich, NR4 7UH UK; 3 Present Address: Synthace, The WestWorks, 195 Wood Lane, 4th Floor, London, W12 7FQ UK; 4 Present Address: Department of Biosciences, Durham University, Stockton Road, Durham, DH1 3LE UK

**Keywords:** Single photons and quantum effects, Quantum optics, Computational science

## Abstract

Oilseed rape (*Brassica napus*) is an important global oil crop, with spring and winter varieties grown commercially. To understand the transcriptomic differences between these varieties, we collected transcriptomes from apex and leaf tissue from a spring variety, Westar, and a winter variety, Tapidor, before, during, and after vernalisation treatment, until the plants flowered. Large transcriptomic differences were noted in both varieties during the vernalisation treatment because of temperature and day length changes. Transcriptomic alignment revealed that the apex transcriptome reflects developmental state, whereas the leaf transcriptome is more closely aligned to the age of the plant. Similar numbers of copies of genes were expressed in both varieties during the time series, although key flowering time genes exhibited expression pattern differences. *BnaFLC* copies on A2 and A10 are the best candidates for the increased vernalisation requirement of Tapidor. Other *BnaFLC* copies show tissue-dependent reactivation of expression post-cold, with these dynamics suggesting some copies have retained or acquired a perennial nature. *BnaSOC1* genes, also related to the vernalisation pathway, have expression profiles which suggest tissue subfunctionalisation. This understanding may help to breed varieties with more consistent or robust vernalisation responses, of special importance due to the milder winters resulting from climate change.

## Introduction

Plants change growth and development in response to their environment^1^. Different varieties of plants have evolved different life strategies, flowering and setting seed at particular times of the year to adapt to their surroundings^2^. This is of particular economic importance for crop species, allowing growers to choose varieties well suited to the climate and their cropping schedule; oilseed rape (OSR; *Brassica napus*), for instance, has spring and winter varieties that are both grown commercially^3^. Understanding how signals from the environment are integrated by the plant is important to breed new crop varieties with favourable characteristics, particularly given the projected effects of unpredictable temperature variation on OSR yield^4^.

Flowering time is a key adaptive trait that is affected by climate^5^. The timing of the floral transition, where the apex transitions from vegetative to floral growth, is one component which affects the final flowering time of a plant. Control of the floral transition depends on multiple environmental cues, such as photoperiod and temperature, which are integrated with internal signals to determine when the plant should flower^6^. The flowering of some plants is accelerated by experiencing an extended period of cold temperature, which is called vernalisation^7^. In the field, such plants can be planted before winter and the vernalisation requirement ensures that the plants do not flower until spring. In the model species *Arabidopsis thaliana* (Arabidopsis), the delay of flowering in the absence of vernalisation is due to expression of *FLOWERING LOCUS C* (*FLC*)^8,9^. *FLC* delays flowering by repressing expression of the flowering activator *FT*^10,11^. *FT* is downstream of both the vernalisation pathway, via *FLC*, and the photoperiod pathway, which senses day length^12–15^. As external signals are sensed in different plant organs, communication between them is important for the signals to be properly integrated into a coherent gene regulatory response. *FT* is expressed in the phloem companion cells, with the FT protein transported in the plant vasculature from leaves to the apex to promote flowering^16–19^. A gene which acts antagonistically to *FT* in the floral transition is *TERMINAL FLOWER 1* (*TFL1*), which represses the floral state of the shoot apical meristem^20–22^. *TFL1* and *FT* are closely related proteins^23^ and both bind DNA in complex with the bZIP transcription factor FD^24,25^. As a result, both influence the expression of similar sets of genes in the gene regulatory network controlling flowering^26^. *SUPPRESSOR OF CONSTANS 1* (*SOC1*) is also a floral integrator downstream of the photoperiod and vernalisation pathways, directly downstream of *FLC*^10,27–29^. In addition, roles have been found for *SOC1* in several other flowering pathways, such as the gibberellic acid pathway^30^, intermittent cold-sensing pathway^31^, and the age-dependent pathway^32^.

Although both members of the Brassicaceae family, the direct application of Arabidopsis genetic understanding to OSR is not straightforward. *Brassica napus* is an amphidiploid derived from ancestral allopolyploidisation of *B. oleracea* and *B. rapa,* related mesohexaploids^33–35^. This has resulted in multiple copies of many genes in OSR that are represented as single copies in Arabidopsis^36^*,* with flowering time genes overrepresented among those genes retained^37^. Gene multiplication provides opportunities for gene neo- and subfunctionalisation, for example, in tissue-specificity, in response to stimuli, and in location in the gene regulatory network^38^. Despite these challenges, knowledge of the floral pathways in Arabidopsis has been translated to Brassica crops^36,39^. In the same way as Arabidopsis, there is variation in the vernalisation requirement between OSR varieties. Indeed, whether a variety is a spring variety (sown in the spring and harvested in the same year) or a winter variety (sown in the summer or autumn and harvested the following year) is a commercially relevant trait, with winter varieties being preferred in Europe and Asia and spring varieties grown in Australia, Canada, and northern Europe^3^. This variation in vernalisation requirement has been found to be associated with copies of *BnaFLC*^40–42^, with *BnaFLC* copies on chromosome A10 and A2 exhibiting the strongest association^42–44^, although variation across multiple *BnaFLC* copies have been found to drive diversity within *Brassica* crop types^45^. *BnaFT* genes in OSR have also been found to exhibit different expression patterns, with variation in the effect on flowering time present between copies^46,47^. In particular, the *BnaFT.A2* gene is strongly associated with flowering time differences between two winter OSR varieties^44^. Within other flowering time integrators, a specific promoter variant of *BnaSOC1.A5* is predominantly found in winter OSR varieties^48^. These findings point towards variation in known flowering time genes from Arabidopsis being responsible for flowering time diversity between OSR, particularly in terms of vernalisation requirement.

Perhaps because of the known roles of OSR homologues of Arabidopsis flowering time genes in determining flowering time, many previous transcriptomic studies in OSR have tended to focus on individual gene copies^49–51^. However, understanding the similarity of transcriptomes on a global level allows for more general expression dynamics to be elucidated^52^. To determine if gene regulation is coordinated between leaves and the growing apex during the floral transition in OSR, we collected a transcriptomic time series from the first true leaf and apex across development. To understand how vernalisation affects gene expression in both tissues, we sampled tissue from both a winter OSR variety, Tapidor, and a spring OSR variety, Westar. Comparisons between the transcriptomes between tissues suggests that transcriptomic changes in the apex are aligned to the developmental state of the plant, whereas in the leaf the transcriptomic changes are aligned based on the age of the plant. This is reflected by the apex transcriptome exhibiting large changes during flower development, whereas the leaf transcriptome changes coincide with leaf senescence. Comparing between Westar and Tapidor, we find two copies of *BnaFLC* (*BnaFLC.A2* and *BnaFLC.A10*) as being the best candidates for mediating the stronger vernalisation requirement of Tapidor relative to Westar. Copies of *BnaSOC1*, a key floral integrator, exhibit expression patterns which are dependent on tissue, suggesting that copies have undergone sub-functionalisation.

## Results

### Developmental differences between varieties are reflected in the apex transcriptomes, but not the leaf transcriptomes

**Figure 1:**
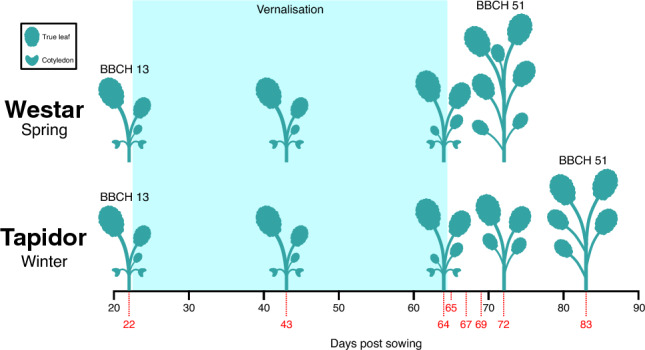
Cartoon of the developmental stages of Tapidor and Westar at each time point. Plant tissue was sampled on the days indicated by red dotted lines and numbers. The plant silhouettes represent the approximate number of full leaves at the indicated points in development, allowing the developmental stage of the plants to be estimated. On day 69, only Westar apex samples were sequenced.

To understand the transcriptomic differences underlying the different flowering strategies of Tapidor, a winter OSR variety, and Westar, a spring OSR variety, the apex and the first true leaf were sampled across development, from 22 days post sowing to when floral buds were visible, for both varieties (Fig. 1). Plants from both varieties were subjected to a cold treatment, which began 23 days post sowing and ended after day 64 of the time series. Although Westar, the spring variety, does not have a vernalisation requirement, it does exhibit a vernalisation response^53^. Therefore, subjecting both varieties to the same treatment allows for clearer comparisons to be made between them. Transcriptomic time series were generated for both varieties and both tissues^37,54^. To ensure that comparable periods of development were captured between varieties, the BBCH scale was used to assess developmental stage^55^. At the first time point sampled (22 days post sowing), plants of both varieties were at BBCH stage 13 (3 leaves unfolded), but subsequent development deviated in time: Westar plants reached BBCH stage 51 (Flower buds visible from above) at day 72 of the time series, whereas Tapidor plants reached the same developmental stage at day 83.

**Figure 2:**
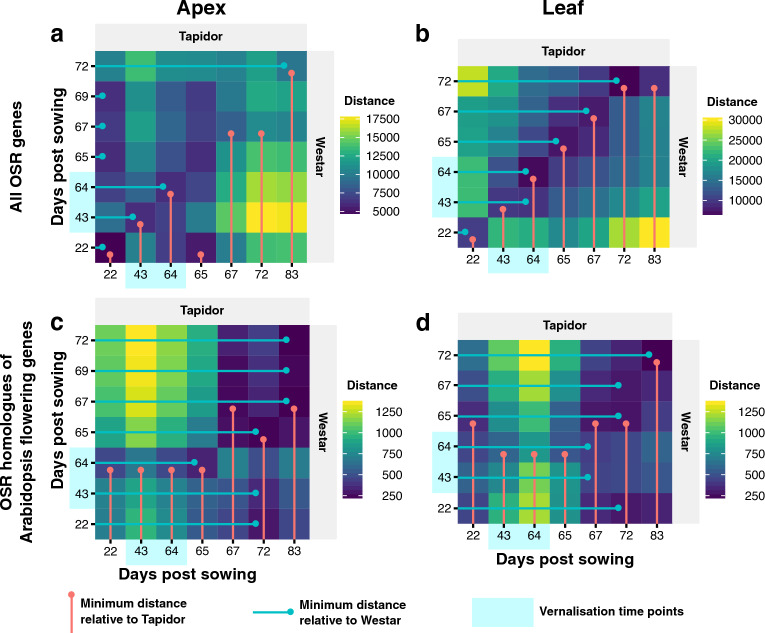
Flowering genes show a different time alignment compared to all OSR genes. Euclidean distances were calculated between the time points from different varieties for both the apex (**a, c**) and leaf (**b, d**). The distances were calculated using all OSR genes (**a, b**) and only those OSR genes showing homology to Arabidopsis genes which are in the FLOR-ID database^56^ (**c**, **d**). The minimum distances are shown by coloured points and lines, with the vertical, red lines showing, for each Tapidor time point, the Westar time point with the smallest distance to it, and the horizontal, blue lines showing, for each Westar time point, the Tapidor time point with the smallest distance to it. The two time points collected during the vernalisation treatment (day 43 and 64) are indicated with a blue background.

To evaluate whether these differences in development were reflected in global gene expression changes, we compared the transcriptomes over time using the Euclidean distance (Materials and Methods). The maximum similarity between transcriptomes can be visualised by the lines overlayed on the distance heatmaps, which indicate the minimum distance relative to each time point (Fig. 2). Using all OSR gene models (155,240) to calculate the distances, the first time point for both tissues is very similar between varieties, as are the transcriptomes sampled during the cold treatment on days 43 and 64 (Fig. 2a,b). After cold, however, the minima diverge in the two tissues. In the apex, the Westar time points post-cold are most similar to the day 22 time point in Tapidor, except for the final time point which is most similar to the final time point in Tapidor (blue, horizontal lines; Fig. 2a). For Tapidor, while the first time point post-cold is most similar to the first Westar time point (day 22), the day 67 and 72 time points in Tapidor are most similar to the day 67 time point in Westar, with the final time points once again being most similar, despite being sampled on different days (red, vertical lines; Fig. 2a). This is consistent with the developmental differences observed between plants (Fig. 1). The most similar time points between varieties in the leaf are those sampled at the same time, with the day 83 time point in Tapidor being most similar to the day 72 time point in Westar (Fig. 2b). These results show that the best agreement between the leaf transcriptomes of these two varieties is aligned with time (age), whereas the apex samples show a more complex relationship dictated by developmental stage.

To determine whether these results were affected by the different flowering regimes of the two varieties, we calculated the distances over time between a subset of 1380 OSR genes that showed homology to Arabidopsis flowering genes, defined as those genes included in the FLOR-ID database^56^. The FLOR-ID database is a list of 346 Arabidopsis genes which have been well characterised in the literature as being involved with flowering or influencing flowering time. The age alignment observed between the global leaf transcriptomes is not seen in the set of flowering genes (Fig. 2d). This suggests that the differences observed between tissues at the global scale are not defined by floral genes. For both tissues, the Westar samples all show highest similarity (lowest Euclidean distance) with Tapidor samples taken after the cold treatment (blue, horizontal lines; Fig. 2c,d). The early Tapidor samples (day 22 to 65) show highest similarly to the day 64 and 65 time points in Westar, which correspond to the final day of cold treatment and first day post-treatment respectively. Given that the cold treatment will have blocked flowering in the Westar plants, this is consistent with flowering being repressed in the Tapidor plants prior to being vernalised. There is also a marked block of similarity in both tissues, beginning on day 67 of the Tapidor time series and day 65 of the Westar time series. This similarity starts immediately after the vernalisation treatment in Westar, and after a slight delay after the vernalisation treatment in Tapidor. These findings suggest that the Westar plants were competent to flower at the very beginning of the time series, a state the Tapidor plants only reached after cold treatment, consistent with the vernalisation requirements of the two varieties.

### Within the leaf samples, there are more genes expressed specifically in Tapidor

**Figure 3:**
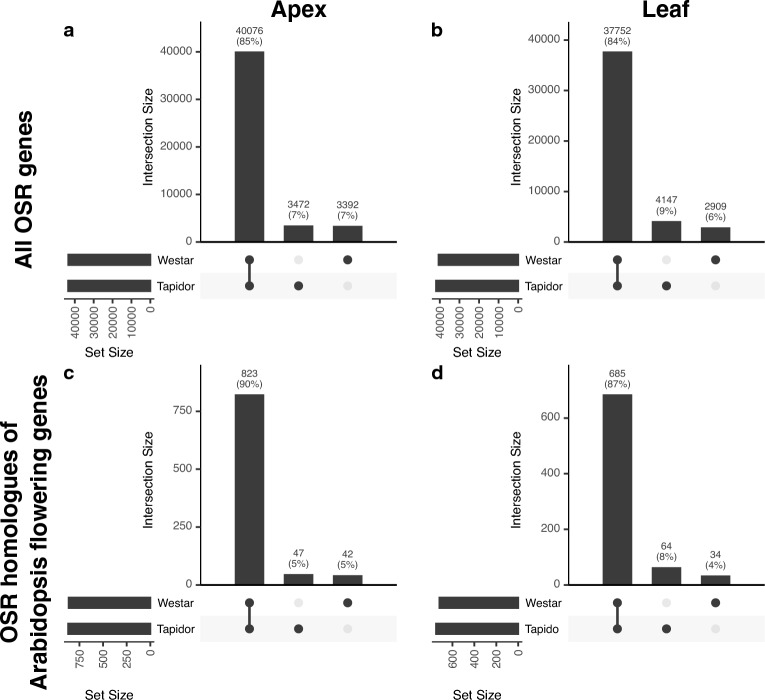
There is high overlap between varieties in the sets of expressed genes. OSR genes were regarded as expressed if their maximal expression level across the transcriptomic time series was greater than, or equal to, 2.0 FPKM. The gene subsets used to calculate the overlaps in each case are: (**a, b**) All OSR genes; (**c, d**) OSR genes that show sequence similarity to Arabidopsis genes in the FLOR-ID database of floral genes^56^. Percentages have been rounded to the closest integer.

We next assessed gene level expression differences between Tapidor and Westar. To do this, all 155,240 OSR genes were classified as expressed if the maximal expression level of the gene in at least one time point across the time series was equal to or exceeded 2.0 FPKM. We find that the vast majority of expressed genes (37,752 in the leaf and 40,076 in the apex, representing 84% and 85% of the total number of expressed genes considered respectively) are expressed in both varieties (Fig. 3a,b). This overall pattern is maintained even when only the 1380 OSR flowering genes (Fig. 3c,d) and 79,014 OSR genes with Arabidopsis homologues (Supplementary Fig. 1) are considered. The number of genes that are specifically expressed in each variety is similar in the apex for all subsets of genes considered (Fig. 3a,c), but in the leaf there are consistently more genes specifically expressed in Tapidor relative to Westar (4147 versus 2909 for all OSR genes and 64 versus 34 for OSR flowering genes; Figs. 3b,d).

To functionally characterise the variety-specific genes, gene ontology (GO) term enrichment was conducted (Supplementary Tables 1 and 2). In the apex, the Westar-specific genes were enriched for defence related GO terms (GO:0009751 response to salicylic acid; GO:0031348 negative regulation of defence response), whereas the Tapidor-specific genes were enriched for terms related to nitrogen and ion transport (GO:1901698 response to nitrogen compound; GO:0006820 anion transport). In the leaf, the Westar-specific genes did not show high enrichment (*p*-value < 10^–5^) for any term, while the Tapidor-specific genes were enriched for DNA replication terms (GO:0006275 regulation of DNA replication; GO:0006260 DNA replication).

**Figure 4:**
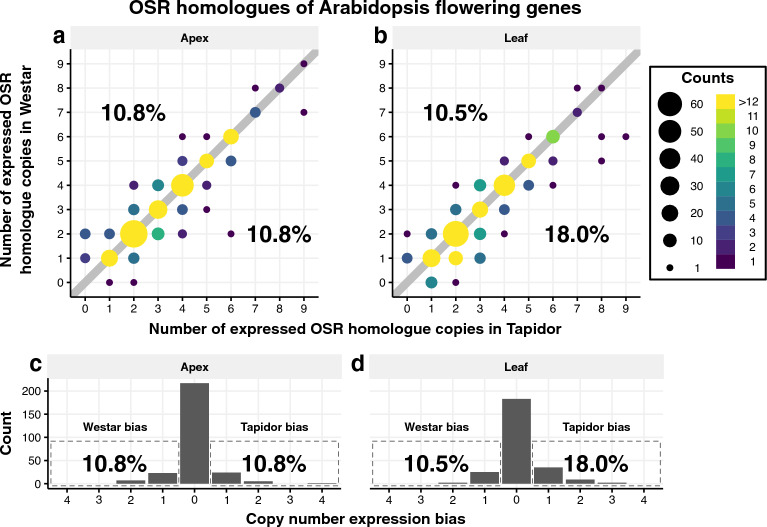
The majority of Arabidopsis floral genes have the same number of expressed OSR orthologues in each variety. (**a, b**) OSR homologue families were defined as OSR genes which have the same Arabidopsis gene as their highest scoring BLAST hit. Expressed OSR genes were determined as those that had a maximal expression value above or equal to 2.0 FPKM at one or more time points in the time series. The size and colour of the circles indicate the number of data points at that position. The upper limit of the colour scale is the maximal off-diagonal value. Points on the diagonal, grey line represent OSR homologue families that have equal numbers of homologues expressed in both Tapidor and Westar. The left most percentage within each graph represent the percentage of Arabidopsis genes that have more homologues expressed in Westar, whereas the right most percentage is the corresponding percentage for Tapidor. (**c, d**) Bar charts sum the number of data points on the diagonals in **a** and ** b**, with copy number expression bias determined as the absolute difference between the number of expressed genes in a OSR homologue family in both Tapidor and Westar.

The higher number of OSR flowering genes expressed only in Tapidor, relative to genes expressed only in Westar, may be due to certain sets of homologous genes. To assess this, OSR flowering genes were first grouped into homologue families based on their sequence similarity to genes in Arabidopsis. Then, the number of expressed copies within each OSR homologue family were compared between Tapidor and Westar (Fig. 4). If genes lie on the diagonal line in Fig. 4a,b, it indicates that the same number of gene copies are expressed in both Tapidor and Westar, while deviation from that line shows that more copies are expressed in either Tapidor (below the line) or Westar (above the line). This data is also represented as count data of the number of genes exhibiting copy number expression bias (Fig. 4c,d). For example, a gene with five copies expressed in Tapidor and three copies expressed in Westar would be said to have a two copy bias towards Tapidor. A striking observation, unrelated to copy number expression bias, is that 47.7% of OSR flowering gene homologue families have two or four copies expressed in the apex in both varieties (44.5% of families in the leaf). This is likely due to the hybridisation of the A and C genomes in OSR, resulting in a bias for an even number of expressed copies in both varieties (Fig. 4a,b). In the apex, the same percentage of homologue families exhibit variety-specific bias towards Tapidor and Westar (10.8%; Fig. 4a,c). However, in the leaf there are significantly (*p*-value = 0.03442; binomial test) more sets of homologue families in Tapidor (18.0%) relative to Westar (10.5%; Fig. 4b,d). Percentages of homologue families exhibiting copy number expression bias (Fig. 4) are higher than the percentages of OSR flowering genes exhibiting variety-specific expression (Fig. 3). For example, 10.5% of homologue families have more genes expressed in the leaf in Westar relative to Tapidor (Fig. 4b), whereas 4% of OSR flowering genes are expressed specifically in Westar (Fig. 3b). This suggests that OSR flowering genes exhibiting variety-specific expression are generally well distributed among different homologue families, rather than the pattern being the result of relatively few families. The same pattern, albeit marginally less strong, is also observed when all 79,014 OSR genes with an Arabidopsis orthologue are compared (Supplementary Fig. 2). A scenario that is not captured by these plots is compensatory orthologue expression. That is, gene copies with Tapidor-specific expression are compensated for by copies with Westar-specific expression, resulting in the gene still being located on the diagonal in Fig. 4 and Supplementary Fig. 2 despite variety-specific expression. While this scenario is observed, the frequency is very low, being observed in just 1.6–2.7% of homologue families, depending on the gene subset and tissue considered (Supplementary Figs. 3, 4).

### Self-organising maps reveal characteristic expression dynamics within the apex and leaf

**Figure 5:**
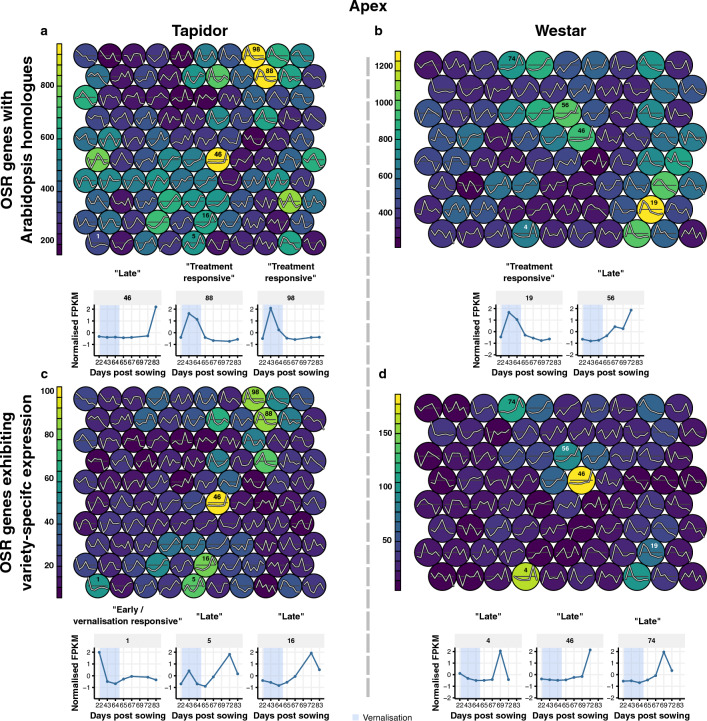
Self-organising maps based on apex transcriptomes reveal that variety-specific genes tend to be expressed at the end of the time series. SOMs generated using either all OSR with Arabidopsis homologues (**a, b**) or OSR genes exhibiting variety-specific expression (**c, d**). (**a, b**) The representative expression traces of the SOM clusters with the most genes assigned to them are plotted below the SOM. (**c, d**) The representative expression traces of the SOM clusters most enriched for variety-specific genes are plotted below the SOM.

To assess the temporal dynamics captured by our dataset, we employed self-organising maps (SOMs) to cluster gene expression profiles (Fig. 5 and Supplementary Figs. 5, 6). Neighbouring SOM clusters have similar expression profiles, with clusters at the opposite boundaries being adjacent to prevent boundary effects. In this way, SOMs represent a toroidal landscape of expression profiles onto which genes are mapped. Selecting the clusters with the most genes mapped to them highlights several distinct expression profile types which will be used to comment on the results. “Early/vernalisation responsive” clusters show high expression initially, with expression decreasing during the vernalisation treatment. This name reflects that, given our experimental setup, genes which decrease in expression across development regardless of cold, and genes which decrease in expression due to being vernalisation responsive, will cluster together. “Treatment responsive” clusters exhibit an increase in expression during the vernalisation treatment, while “Late” clusters peak in expression towards the end of the time series. Finally, “Treatment responsive/Late” clusters consist of expression profiles which show increases in expression during the cold treatment and towards the end of the time series.

For the apex data from Tapidor, the clusters with the most genes mapped to them are “Late” and “Treatment responsive” (clusters 46, 88, and 98, which have 2.3%, 2.4%, and 2.3% of the total number of genes mapped to them respectively; Fig. 5a). The “Late” cluster is highly enriched for genes with the GO terms “carpel development”, “gynoecium development”, and “floral whorl development”. The two “Treatment responsive” clusters are both enriched for genes with the GO term “circadian rhythm”. This is likely due to the change in day length during the vernalisation treatment. Interestingly, cluster 88 is also enriched for genes with the GO terms “starch catabolic process” and “glucan catabolic process”, suggesting a change in nutrient usage during the vernalisation period. A similar pattern is observed in the Westar apex data (Fig. 5b), with the most genes mapping to a “Treatment responsive” cluster (cluster 19, 3.2% of genes mapped), enriched for genes with the GO terms “long-day photoperiodism” and “circadian rhythm”, and a “Late” cluster (cluster 56, 2.5% of genes mapped), enriched with the GO terms “carpel development” and “specification of floral organ identity”.

The expression profiles of the SOM clusters with the most genes mapping to them are also somewhat similar in the leaf samples, although different GO terms are enriched (Supplementary Fig. 6). “Treatment responsive” clusters in both varieties (cluster 25 in Tapidor and cluster 99 in Westar, with 3.2% and 2.0% of the total number of genes mapped to them respectively) are strongly enriched for “translation” and “peptide biosynthetic process” GO terms. The GO term “cell wall modification” is also highly enriched in both clusters, suggesting that the large transcriptional changes observed are related to the leaves acclimatising to the colder growth conditions. The “Late” clusters in both varieties (cluster 59, 3.1% of genes, in Tapidor and cluster 19, 2.4% of genes, in Westar) are strongly enriched for stress, cell death, and age-related GO terms. A potential explanation is given by both clusters also being enriched for “leaf senescence”, which is consistent with the first true leaf being sampled throughout development (Fig. 1).

Mapping the 1380 OSR floral genes to the apex SOMs (Supplementary Fig. 5) reveals the same “Late” and “Treatment responsive” clusters as was seen when all genes are considered. However, for the leaf SOMs, floral genes map to different “Treatment responsive” and “Treatment responsive/Late” clusters (clusters 5, 8, 15, and 67 in Tapidor and clusters 55, 66, and 77 in Westar; Supplementary Fig. 6). Cluster 5 in Tapidor and cluster 55 in Westar are both “Treatment responsive” clusters, and are enriched for the GO term “photoperiodism, flowering”, suggesting that floral genes in the leaf are primarily responding to the change in day length during the vernalisation period.

**Figure 6:**
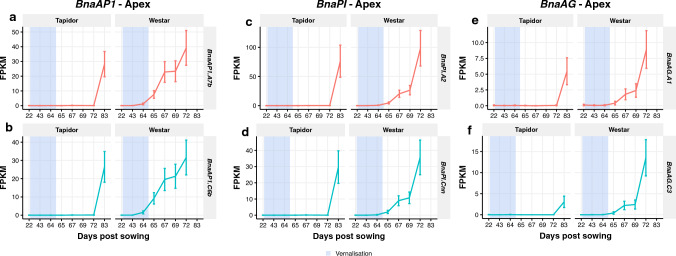
ABC floral genes show alignment with developmental stage of the plant. Expression traces across the developmental time series in apex tissue for representative copies of *BnaAP1* (**a, b**), *BnaPI* (**c, d**), and *BnaAG* (**e, f**). The blue segments indicate the time points sampled during the vernalisation treatment.

The SOM analysis in the apex in both varieties revealed “Late” SOM clusters as being among the clusters with the most genes assigned to them (cluster 46 in Tapidor, with 2.3% of mapped genes, and cluster 56 in Westar, with 2.5% of mapped genes). Both clusters were enriched for genes with GO terms related to flower development. To analyse this with more detail, we looked at the expression of the ABC floral patterning genes, which have been found to specify floral organ development through overlapping and combinatoric gene expression domains^57^ (Fig. 6). OSR homologues of *APETELA1* (*AP1*), *PISTILLA* (*PI*), and *AGAMOUS* (*AG*) all exhibit an expression delay in Tapidor when compared to Westar (Fig. 6). The levels of expression of *BnaAP1* (Fig. 6a,b) and *BnaPI* (Fig. 6c,d) copies in Westar on day 72 of the time series are only reached in Tapidor by the day 83 time point. With the copies of *BnaAG*, equivalent expression levels to Westar are not obtained in Tapidor during the sampled time series (Fig. 6e,f). These temporal differences closely follow the delayed onset of flowering in Tapidor relative to Westar (Fig. 1).

**Figure 7:**
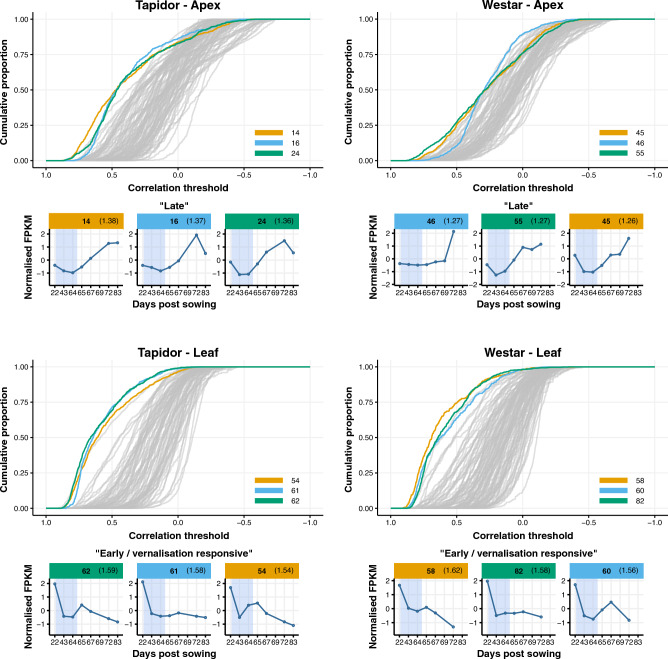
“Late” SOM clusters show greatest correlation to the global expression changes in the apex, whereas "early/vernalisation responsive" clusters show greatest correlation in the leaf. Euclidean distances matrices were created for each gene and compared to the global transcriptome response (Fig. 2) by Pearson correlation coefficient. The relationship between the proportion of genes within each SOM cluster with a correlation coefficient greater than a particular threshold is shown. The representative expression traces of the three SOM clusters with the highest area under this curve (areas reported within brackets in figure headers) are shown below the plots.

We can also use the SOM clusters to summarise which genes correlate most with the global transcriptome distance differences observed between varieties in Fig. 2. Calculating Pearson correlation coefficients between the global and gene-based distance matrices, and using an area under the curve metric, we identified the three SOM clusters, for each variety and tissue, with the highest proportion of genes highly correlated with the global pattern (Materials and Methods; Fig. 7). It should be noted that because we have considered the proportion of genes within each SOM cluster, this will not necessarily represent the genes which most contribute to the global transcriptome differences, but those which are most characteristic of it by correlation. In the apex, three “Late” clusters have the highest area under the curve for both varieties, whereas “Early / vernalisation responsive” clusters have the highest area for the leaf in both varieties. This again supports our finding that the apex is driven by developmental progression towards flowering, whereas the leaves are more affected by environmental changes.

### Tapidor-specific genes in cold-responsive SOM clusters contain known vernalisation genes

To better characterise transcriptome differences between varieties, we tested SOM clusters for enrichment of genes expressed in a variety-specific manner. Fisher tests were used to test each cluster in the SOM for enrichment of genes expressed in a variety-specific manner, relative to all expressed OSR genes in that variety with identified Arabidopsis orthologues (Materials & Methods). The top three clusters showing enrichment, with an adjusted *p*-value below 10^–5^, are labelled (Fig. 5c,d; Supplementary Table 3). Across both varieties and tissues, the variety-specific genes tend to be enriched in “Late” clusters (Fig. 5c,d; Supplementary Fig. 6). This suggests that the expression profiles of the varieties diverge towards the final time points. In addition, the genes expressed specifically in Tapidor are also enriched in “Early/vernalisation responsive” clusters in both tissues. This enrichment is not seen in Westar and mimics the expression pattern of documented vernalisation responsive genes, raising the possibility that some of the Tapidor-specific expressed genes mediate, or are downstream of, the vernalisation response in Tapidor.

As differential enrichment of “Early/vernalisation responsive” clusters was observed, we investigated the genes mapped to these clusters in more detail (Supplementary Table 4). In terms of Tapidor-specific expressed genes mapped in the apex (Cluster 1; Fig. 5c) we find two orthologues of *FLOWERING LOCUS C* (*FLC*) as the only two annotated flowering time genes (*BnaFLC.A10* and *BnaFLC.C3c*. *BnaFLC.C3c* is a split model for *BnaFLC.C3b*^45^; model details for genes discussed by name are given in Supplementary Table 5). In the Tapidor-specific expressed genes mapped in the leaf (Cluster 61; Supplementary Fig. 6c) we find four orthologues of *FLC* (*BnaFLC.A3b, BnaFLC.A10, BnaFLC.C3a,* and *BnaFLC.C3c*) and one orthologue of *MADS AFFECTING FLOWERING 4* (*MAF4*; *BnaMAF4.A2a*) as the only annotated flowering genes, all of which are documented vernalisation responsive genes in Arabidopsis and *Brassica* species^58–60^.

We then expanded our focus beyond genes which are expressed in a variety-specific manner in these clusters but continued to restrict the list of genes to annotated flowering genes. Doing so identifies an additional three *BnaFLC* genes (*BnaFLC.A2*, *BnaFLC.A3a*, and *BnaFLC.C3b*), two OSR orthologues of *AUXIN RESPONSE TRANSCRIPTION FACTOR 3*, one OSR orthologue of *PSEUDO-RESPONSE REGULATOR 7*, and one OSR orthologue of *FT-INTERACTING PROTEIN 1*. Similarly, in the leaf, an additional three *BnaFLC* genes are clustered to cluster 61 but do not exhibit variety-specific expression (*BnaFLC.A2, BnaFLC.A3a*, and *BnaFLC.C2*). OSR orthologues of *PHOSPHOGLUCOSE ISOMERASE 1, EARLY FLOWERING MYB PROTEIN*, *APETALA 2,* and *GIBBERELLIN 20-OXIDASE 3* are also annotated as flowering genes and mapped to cluster 61.

### *BnaFLC* gene expression reveals copy-specific differences in expression level between Tapidor and Westar and different reactivation dynamics between apex and leaf

**Figure 8:**
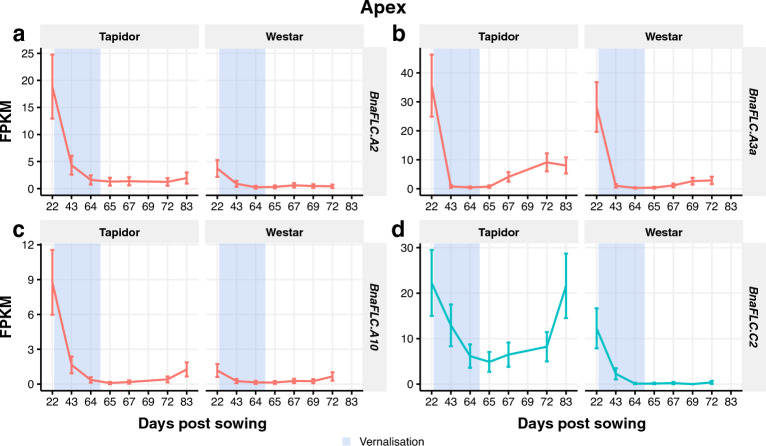
*BnaFLC.A2* and* BnaFLC.A10* genes appear to be the primary copies of *BnaFLC* mediating the vernalisation response, based on expression in the apex. Expression traces across the developmental time series in apex tissue for selected copies of *BnaFLC*: *BnaFLC.A2* (**a**), *BnaFLC.A3a* (**b**), *BnaFLC.A10* (**c**), and *BnaFLC.C2* (**d**). The blue segments indicate the time points sampled during the vernalisation treatment.

Two copies of *BnaFLC* were found to be expressed only in Tapidor in the tissues we investigated, and additional copies of *BnaFLC* are mapped to SOM clusters enriched for genes expressed specifically in Tapidor. Given the key role *FLC* plays in the vernalisation response in Arabidopsis and *Brassica* species, we decided to look in more detail at the expression dynamics of the various *BnaFLC* copies and how they differ between the winter (Tapidor) and spring (Westar) variety. We observed qualitatively similar expression responses for each *BnaFLC* copy in both the apex and the leaf (Fig. 8 and Supplementary Fig. 7). All copies except *BnaFLC.C9b* exhibit a decrease in expression during the cold treatment, often in both varieties. Only *BnaFLC.A3a* has a similar expression profile and expression level in both varieties and is expressed to an appreciable level (Fig. 8b). For other copies (*BnaFLC.A2*, *BnaFLC.A3b, BnaFLC.A10, BnaFLC.C2,* and *BnaFLC.C3c*) the expression of the gene in Westar is considerably less than in Tapidor. This expression difference is most prevalent at the beginning of the time series, due to decreasing gene expression levels during the cold treatment. However, for some copies, a reactivation of the gene is observed. Copies of *BnaFLC.A3b* (Supplementary Fig. 7), *BnaFLC.C2* (Fig. 8d), and *BnaFLC.C3c* (Supplementary Fig. 7) in the apex all decrease in expression during the cold treatment, but in Tapidor the expression of the genes increase post-cold. The reactivation is not observed in Westar. Differences in reactivation post-cold also define the major differences in expression profiles between apex and leaf, with *BnaFLC.A2* (Fig. 8a) and *BnaFLC.A10* (Fig. 8c) exhibiting expression reactivation in Tapidor leaf samples yet show more stable repression in the Tapidor apex samples.

The two copies of *BnaFLC* on chromosome C9 show higher expression in Westar rather than Tapidor (Supplementary Fig. 7). The expression of *BnaFLC.C9b* is interesting given that it is highly expressed in both varieties, and counter to the other copies of *BnaFLC* increases in expression over the time series sampled here. There are an additional two *BnaFLC* copies on chromosome C3, which are both very lowly expressed in these two varieties and across this period of development (Supplementary Fig. 7).

### *BnaSOC1* genes exhibit tissue-specific subfunctionalisation in terms of expression

**Figure 9:**
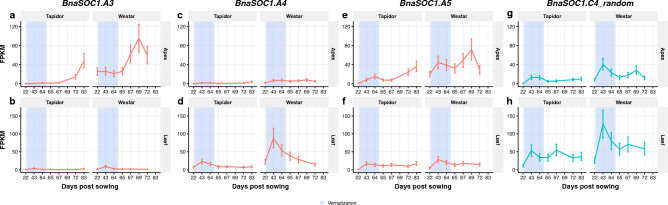
*BnaSOC* genes have undergone subfunctionalisation, resulting in copies which are more prevalent in particular tissues. Expression traces across the developmental time series in apex (**a, c, e, g**) and leaf (**b, d, f, h**) tissue for selected copies of *BnaSOC1*: *BnaSOC1.A3* (**a**, **b**), *BnaSOC1.A4* (**c**, **d**), *BnaSOC1.A5* (**e**, **f**), and *BnaSOC1.C4_random* (**g**, **h**). The blue segments indicate the time points sampled during the vernalisation treatment.

*SOC1* is involved with integration of inputs from an array of different flowering time pathways, including vernalisation^61^. Due to these many functions in the control of flowering, we investigated the expression of OSR *SOC1* homologues in both Westar and Tapidor, to try and unpick the effects of the vernalisation pathway on the expression of *BnaSOC1* genes. We found that the expression of *BnaSOC1* genes was consistently lower in Tapidor compared to Westar across the time series. This is consistent with the repression of *BnaSOC1* genes by *BnaFLC* genes, as in Arabidopsis (Fig. 9). The expression of some copies shows tissue-specificity. Compared to other *BnaSOC1* genes expressed in the apex, *BnaSOC1.A3* shows very high expression (Fig. 9) and increases in expression concurrently with the homologues of the ABC floral genes (Fig. 6). However, in the leaf this copy is lowly-expressed relative to the other *BnaSOC1* genes. Conversely, the A4 copy of *BnaSOC1* is among the most highly expressed copies in the leaf, but is lowly expressed in the apex. While the patterns are more subtle, a similar tissue preference can be observed in the A5 and the C4 copies of *BnaSOC1*. In addition, the copies showing leaf and apex expression preference also exhibit different expression patterns. The genes with a stronger apex preference (*BnaSOC1.A3* and *BnaSOC1.A5*) both reach their highest expression level post-cold and follow the patterns of the ABC floral genes (Fig. 6). However, the genes with a leaf expression preference peak in expression during the vernalisation period. These results all suggest that the *BnaSOC1* genes have undergone sub-functionalisation, with some genes retaining apex-specific roles and others leaf-specific functions. This is consistent with the varied functions identified for *SOC1* in Arabidopsis^10,27–32^ and might reflect the roles *SOC1* plays in the vernalisation, photoperiod, and the intermittent cold-sensing pathways.

### *BnaFT* genes show large expression differences between varieties, and suggest that flowering in Westar was delayed by the cold treatment

**Figure 10:**
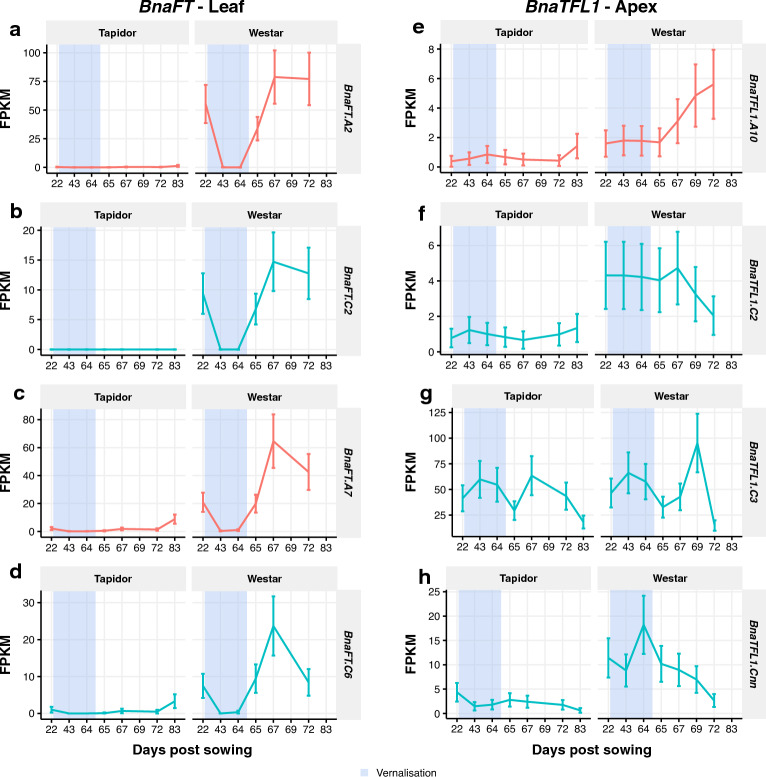
*BnaFT* and* BnaTFL1* genes tend to be more highly expressed in Westar relative to Tapidor, although *BnaTFL1* genes exhibit qualitatively similar expression patterns in both varieties. Expression traces across the developmental time series in leaf for *BnaFT* genes (**a-d**) and apex for *BnaTFL1* genes (**e–h**). The blue segments indicate the time points sampled during the vernalisation treatment.

In Arabidopsis, the effect of *FLC* on flowering time is largely mediated by the FLC protein repressing the expression of *FT* in the leaves^10,11^. To determine if a similar mode of action was taking place in OSR, we looked at the expression of *BnaFT* genes. All four copies are very highly expressed in the leaf in Westar, decreasing to very low expression only during the cold treatment (Fig. 10a–d). This is likely due to the change in day length during the vernalisation period. This effect was seen in the SOM analysis of the leaf (Supplementary Fig. 6) with clusters enriched in the GO term “photoperiodism, flowering” increasing in expression during the cold treatment (Supplementary Table 6). This is expected given the role *FT* plays in sensing day length in Arabidopsis^16–19^. In Tapidor, *BnaFT.A2* and *BnaFT.C2* are very lowly expressed throughout the time series. *BnaFT.A7 and BnaFT.C6* are also lowly expressed, although increase towards the end of the time series, but not to the same extent as the genes in Westar.

*FT* and *TFL1* act antagonistically in the gene regulatory network underlying flowering in Arabidopsis^26^. In our previous work we highlighted the divergence of the *BnaTFL1* genes in OSR, which correlated with the presence and absence of regulatory sequences upstream of the gene^37^. Comparing the expression of the *BnaTFL1* genes between varieties (Fig. 10e–h), we see that the most highly expressed copy on C3 shows a very similar expression profile in both varieties in the apex (Fig. 10g). The other copies, while showing similar expression profiles, are more lowly expressed in Tapidor relative to Westar throughout the time series, with the copies on A10 and C2 being below 2.0 FPKM throughout the time series (Fig. 10e,f).

## Discussion

The pathways controlling flowering in eudicots have been primarily characterised in Arabidopsis. Our use of both a winter and a spring variety of OSR, and the short-day vernalisation treatment, allowed us to investigate how the flowering pathways function in *B. napus*. In particular, it seems that the photoperiod flowering pathway was not inhibiting flowering in Westar plants before the cold treatment. This is evidenced most clearly when looking at individual floral genes. At the first time point, the expression of *BnaFT* genes in the leaves in Westar is high (Fig. 10a–d), yet the expression of *BnaAP1* genes, used as an indicator of the floral transition^26,62^, is low in the apex at the same time (Fig. 6c,d). This is indicative of the plants being grown in a photoperiod conducive to flowering but having not yet transitioned from vegetative to floral growth. The floral transition was then inhibited by the transfer to short-day conditions during the vernalisation treatment, which is again represented by the expression of *BnaFT* genes decreasing to near-zero in the Westar leaf samples (Fig. 10a–d). This is in line with the photoperiod control of *FT* in Arabidopsis^12–14^ and the results from our SOM analysis, which indicate that circadian rhythm and photoperiodism genes are enriched in “treatment responsive” clusters (Fig. 5).

Further evidence that the Westar plants were capable of flowering throughout the time series are the flowering gene-specific distance heatmaps. All Westar time points were found to be most similar in expression to Tapidor time points sampled after the vernalisation treatment (Fig. 2c,d). Tapidor has a facultative, rather than obligate, vernalisation requirement, meaning that vernalisation speeds up the onset of flowering in Tapidor, rather than being a prerequisite of flowering. Cold treatment therefore causes the plants to become competent to flower earlier in development than they would have without the treatment^41^. The observed similarity in Westar samples and Tapidor samples post-cold treatment is therefore consistent with Westar plants being capable of flowering throughout the time series, with Tapidor becoming competent to flower after the cold treatment. Given that the Tapidor plants had visible flower buds at the final time point sampled, it is perhaps surprising that the *BnaFT* genes in the Tapidor leaf samples do not reach the same expression levels as observed in the Westar leaf samples (Fig. 10a–d). Potentially the low expression of *BnaFT* genes in the Tapidor leaf samples is a consequence of the age of the leaves, raising the possibility that the ability of leaves to signal within the photoperiod flowering pathway decreases over time. However, a similar transcriptome time series found that expression of the age-dependent pathway factor, *SPL5*, also increases during vernalisation in a winter variety of OSR, suggesting that this pathway may also be contributing to the floral transition in Tapidor^50^.

The global gene expression differences between varieties suggest that the apex transcriptome reflects floral development, whereas the leaf transcriptome reflects the age of the plant. The distance heatmaps suggest this, with the leaf transcriptomes between varieties tending to be most similar on the same sampling day (Fig. 2b). While this is true of the beginning of the apex time series, the trajectories diverge after the vernalisation treatment; the final time points, sampled when both varieties had reached BBCH stage 51 (Fig. 1), are most similar despite being sampled on different days (Fig. 2a). This conclusion is supported by the GO term enrichment for the most populated “Late” SOM clusters, with floral development terms enriched in those clusters in the apex (Fig. 5 and Supplementary Table 6) and leaf senescence and age-related terms enriched in the leaf (Supplementary Fig. 6 and Supplementary Table 6). This finding is consistent with the results of a study comparing two rapid-cycling *B. rapa* varieties, with gene expression in the first true leaf synchronised over time and gene expression in apex tissue following floral development more closely^52^. The authors suggested that differences in the rate of development between the varieties was likely to be the result of changes at the shoot apex, rather than the leaf, which also seems to be the case with the OSR varieties tested in this study.

Findings from the global level analysis are also observed in the behaviour of key floral genes. The synchronisation of the apex transcriptome with flower development can be seen when looking at copies of the ABC floral genes *BnaAP1*, *BnaPI*, and *BnaAG* (Fig. 6). The expression of these genes in the apex, which can be seen as direct evidence of the floral transition having taken place, begins to increase on day 65 in Westar, whereas expression of these genes is not detected in Tapidor until the final time point at day 83 (Fig. 6). The flowering gene distance heatmaps show that the vernalisation treatment results in the Tapidor and Westar transcriptomes becoming more similar in both tissues (Fig. 2c,d). In Arabidopsis, the expression of *FLC* is a readout of the vernalisation state of the plant; if *FLC* is stably silenced after cold treatment then the plant has been vernalised, while rapid-cycling accessions of Arabidopsis have low levels of *FLC* throughout development primarily due to loss of activity of the *FRIGIDA* gene^8,63^. Looking at the *BnaFLC* genes in OSR, we see that two genes follow this pattern of expression: *BnaFLC.A2* and *BnaFLC.A10*. This is in line with previous findings, with the A2 and A10 homologues of *FLC* being found multiple times to be associated with flowering time in OSR and *B. rapa*^40–42, 44, 50, 64–67^. In Westar, *BnaFLC.A10* contains a LINE insertion and *BnaFLC.A2* contains a hAT retrotransposon, making both genes non-functional^68^. Therefore, despite both genes being lowly expressed at the beginning of the time series, the genes are unlikely to be having any repressive effect on flowering. Although the repression of *BnaFLC.A2* and *BnaFLC.A10* is stable in the apex in Tapidor (Fig. 8a,c), the same is not true in the leaf (Supplementary Fig. 7). This may reflect the number of cells dividing in both tissues. In the meristem of the apex, the more frequent cell divisions may lead to more stable repression compared to the first true leaf^69,70^. However, this explanation does not fully explain the stability of *BnaFLC* gene expression, as other gene copies, such as *BnaFLC.A3b*, exhibit reactivation post-cold in the apex (Supplementary Fig. 7).

Looking to the other copies of *BnaFLC*, we find *BnaFLC.A3b* (Supplementary Fig. 7) and *BnaFLC.C2* (Fig. 8d) are both more highly expressed in Tapidor at the beginning of the time series, although show an expression reduction during the vernalisation treatment in both varieties. While this expression remains low in Westar, in Tapidor there is reactivation post-cold. Reactivation is observed in some Arabidopsis *FLC* alleles when not given adequate cold treatment^71–73^. This may suggest that these particular copies have not received adequate cold in our experiment in order to become fully repressed in Tapidor. In *B. oleracea,* variation in reactivation of *BoFLC.C2* contributes to differences in flowering time in different genotypes^74^. In Arabidopsis, variation in the efficiency of *FLC* silencing contributes to natural variation in vernalisation response^73,75^, with different *FLC* alleles found to have different optimal vernalisation temperatures^71,72^. If this is true for the different *BnaFLC* genes in OSR, with different copies having different optimal vernalisation temperatures, it may allow OSR plants to disentangle the length of vernalisation and the temperatures experienced during cold, to better adjust its development. Alternatively, *FLC* reactivation similar to *BnaFLC.A3b* and *BnaFLC.C2* is observed in the perennial *Arabidopsis halleri*^76,77^. It may be the case that these *BnaFLC* genes exhibiting reactivation retain a perennial mode of action as a result of, or perhaps in spite of, domestication. Another possibility is that the difference between *BnaFLC* genes is an indicator of the vernalisation requirements of the *B. rapa* and *B. oleracea* ancestors that hybridised to form *B. napus* less than 10,000 years ago^33^. However, this is difficult to assess as modern-day *B. rapa* and *B. oleracea* exhibit considerable diversity in their flowering regimes^78,79^ and *FLC* homologue behaviour^80–82^. This is complicated by homologous exchange events, such as the *BnaFLC.C2* gene being replaced by the *BnaFLC.A2* gene in Tapidor, but not in Westar^68^. The array of different *BnaFLC* dynamics observed suggests that these genes have undergone subfunctionalisation. Indeed, in Arabidopsis, the FLC protein was found to bind at hundreds of sites across the genome and, in addition to flowering, potentially have roles in stress and hormone response pathways^83,84^. This also seems to be the case in other Brassicaceae, such as *Arabis alpina*^85^. It is therefore possible that *BnaFLC* genes which were lowly expressed in both varieties in this time series have roles in pathways other than flowering.

In a previous work we showed that *BnaTFL1* genes exhibited very different expression patterns, and this seemed to correspond to differences in the DNA sequence surrounding the gene, which had been found to contain cis-regulatory elements^37^. Comparing between varieties in this study we find the C3 copy of *BnaTFL1* to be highly expressed and have similar expression dynamics in both varieties (Fig. 10g). The other three copies of *BnaTLF1*, however, are more lowly expressed in Tapidor relative to Westar. Further evidence for sub- or neo-functionalisation of flowering time genes comes from comparing *BnaSOC1* genes, which show a clear tissue preference (Fig. 9). This supports other studies which have found *SOC1* homologues in *Brassica* species to have different expression patterns, due to a partitioning of transcription factor binding sites between the different copies^48,86, 87^. There are also between-variety differences between the copies, with the genes being consistently higher expressed in Westar relative to Tapidor (Fig. 9). This may be due to the higher overall *BnaFLC* expression in Tapidor, as in Arabidopsis *FLC* binds within the *SOC1* promoter in vitro and is required for *SOC1* repression in vivo^10,29^.

Many gene-level findings here support those of Matar and colleagues, with similar *BnaFLC* copies exhibiting vernalisation responsiveness and upregulation of *BnaSOC1* genes from reference chromosomes A5 and C4_random in the apex^50^. Where our results diverge, however, are in the expression of *BnaAP1* and *BnaPI* genes, which are expressed during vernalisation in the previous study but expressed after vernalisation in this study (Fig. 6). This may well be due to the longer vernalisation period used (9 weeks compared to 6 weeks) or may represent a physiological difference between both Tapidor and Westar and Express617. Matar et al. also found no significant difference between *BnaSOC1* genes between apex and leaf samples. This is likely due to overall transcript levels being assessed, which would mask the tissue subfunctionalisation we observe here (Fig. 9). Other transcriptomic studies for OSR also exist, investigating differential gene expression between different developmental and vernalisation states^49,51^. Although all these studies focus on OSR homologues of known flowering genes, they often lack more general analysis of global transcriptome dynamics. In this work we show that by calculating Euclidean distances between transcriptomes, and using SOMs, we can observe global dynamics which suggest the apex and first true leaf transcriptomes are both strongly affected by the cold treatment, which appears to be primarily due to the change in day length. This allows gene-level results, such as the downregulation of *BnaFT* genes (Fig. 10) to be considered in context with what is happening at the global level.

In this work we have compared the transcriptomes of two varieties of OSR during pre-floral development. Taking the whole transcriptome into consideration, we find that the apex transcriptome seems to represent the developmental state of the plant, while the first true leaf transcriptome reflects the age of the plant. Within each tissue, however, the transcriptomes between varieties were found to be largely similar. Differences became apparent between varieties when focussing on floral genes, and in particular, *BnaFLC* genes. Additionally, *BnaSOC1* genes appear to have undergone tissue-specific subfunctionalisation. The transcriptomes presented here provide an important resource for OSR researchers investigating flowering related genes, and as such, we have made the data available in a web based expression browser^54^ (order.jic.ac.uk).

## Materials and methods

### Plant growth and sampling

*Brassica napus* cv. Westar and *Brassica napus* cv. Tapidor plants were grown and sampled as previously described^37^. Plants were sown on 7th May 2014 in cereals mix and grown in unlit glasshouses in Norwich, UK, with temperatures set at 18 °C during the day and 15 °C at night. The day length was approximately 16 h. On day 22 plants were transferred to a 5 °C controlled environment room with 8 h days. After 6 weeks at 5 °C, plants were transferred back to the unlit glasshouses and grown until the plants flowered.

The first true leaf and shoot apex of each plant was sampled at 22, 43, 64, 65, 67, 69, 72, and 83 days after sowing (between BBCH stages 13 and 52). To ensure that this developmental range was aligned between varieties, towards the end of the time series varieties were samples on different days from each other (Table 1). The first true leaves were cut and immediately frozen in liquid nitrogen. The shoot apices were dissected on a chilled tile before being transferred to liquid nitrogen.

**Table 1:** Sampling and sequencing scheme used to generate transcriptomic time series.

Date sampled	Days post-sowing	Days vernalised	Days post-vernalisation	Tapidor		Westar	
				Leaf	Apex	Leaf	Apex
2014-05-29	22	0	0	2	2	2	2
2014-06-19	43	21	0	2	2	2	2
2014-07-10	64	42	0	2	2	2	2
2014-07-11	65	42	1	1	1	1	1
2014-07-13	67	42	3	2	2	2	2
2014-07-15	69	42	5	–	–	–	1
2014-07-18	72	42	8	2	2	2	2
2014-07-29	83	42	19	2	2	–	–

The date samples were taken, the number of days post-sowing this date corresponds to, the number of days the samples were vernalised for at that point, and the number of days post-vernalisation are shown for each day samples were taken. The numbers in the rightmost four columns indicate the number of biological pools sampled and sequenced for each tissue on that date. 0.1 g of apices or 6–10 leaves from different plants constituted one pool of samples. Sequencing pools of tissues from different plants averages the biological variability present between plants, while sequencing multiple pools allows for noise from technical and biological sources to be considered.

Samples were pooled and ground in preparation for RNA extraction. For apex tissue, 0.1 g of apices were ground. For leaf samples, between 6 and 10 leaf samples from separate plants were pooled and ground. RNA extraction and DNase treatment were performed following the method provided with the E.Z.N.A® Plant RNA Kit (Omega Bio-tek Inc., www.omegabiotek.com).

Library preparation and RNA sequencing were carried out by the Earlham Institute, Norwich, UK (www.earlham.ac.uk). RNA sequencing (RNA-Seq) was performed on an Illumina HiSeq2500, generating 100-bp single-end reads. The number of biological pools sequenced for each time point, for each tissue, is indicated in Table 1. Gene expression levels were quantified using the previously published approach. Briefly, the Darmor-*bzh* reference genome sequence^88^ was used to align reads to. AUGUSTUS^89^ (version 3.2.2) was used to perform gene model prediction, using the aligned reads to aid intron detection. The Tuxedo suite of software was used to align reads and quantify gene expression levels^90^. As in our previous work, genes were defined as expressed if their maximal expression level across the transcriptomic time series was greater than, or equal to, 2.0 FPKM.

### Floral gene annotation

OSR gene models were assigned homologous Arabidopsis gene names based on a blastn^91^ (version 2.2.30 +^91^) approach already published^37^. OSR floral genes are defined as those gene models with homologous Arabidopsis gene models which are in the FLOR-ID database of flowering genes^56^.

### Euclidean distance calculation

The dist function in the R statistical programming language was used to calculate the Euclidean distance between transcriptomes. Either all genes were used, or just the OSR flowering time genes as defined above.

### UpSet plots of expressed genes

UpSet plots were plotted using the UpSetR package^92^ (version 1.4.0).

### Self-organising map analysis

The self-organising map (SOM) analysis was carried out as previously reported^37^, with SOM ratio set to the ratio of eigenvalues of the first two principal components and the size determined using a sum of squared distance metric. For each tissue and variety combination, all 155,240 OSR genes were filtered to remove any genes not detected in half of time points, any genes not expressed above 2.0 FPKM at any point in the time series, and any genes which did not have an Arabidopsis homologue, in that order. The number of genes removed by each filtering step, and the number of genes clustered for each SOM, is shown in Table 2. The kohonen package^93^ (version 2.0.19) was used to calculate and plot the SOMs.

**Table 2:** Numbers of OSR genes remaining after each filtering step.

Filtering stage	Tapidor		Westar	
	Leaf	Apex	Leaf	Apex
	155,240	155,240	155,240	155,240
1. Genes detected in half of all time points	75,655	76,175	76,672	76,830
2. Genes expressed above 2.0 FPKM in at least 1 time point	41,889	43,399	40,650	43,357
3. Genes with an Arabidopsis orthologue	38,728	40,137	37,616	40,104

Before being clustered in the SOM, the 155,240 OSR genes were filtered to remove genes which did not meet each filtering criteria. The numbers in the table are the numbers of genes remaining after each filtering stage, going from top to bottom. The number of genes in the bottom row represents the final set of genes clustered for each combination of tissue and variety.

### Enrichment of variety specific genes within SOM clusters

For each SOM cluster, a Fisher test was conducted using the fisher.test function in the R statistical programming language to test if variety specific genes were enriched in that cluster relative to the total number of genes mapped to that cluster. The p-values from these tests were False Discovery Rate^94^ adjusted using the p.adjust function.

### Gene ontology analysis

Gene ontology (GO) terms were assigned to OSR gene models based on Arabidopsis GO term assignments. The Arabidopsis gene with the highest BLAST bit score to each OSR gene model was taken, and the Arabidopsis GO assignments for that gene, from the org.At.tair.db package^95^ were assigned to the OSR gene. GO enrichment analysis was performed using the topGO package^96^.

### SOM cluster correlation

For each SOM cluster, between-variety Euclidean distance matrices, such as those in Fig. 2, were calculated for each individual gene. A tissue-specific Pearson correlation coefficient was calculated for each gene by calculating the coefficient between each gene-specific distance matrix and the global distance matrix by flattening the matrices into a vector. Using this method, genes with a high correlation coefficient have a similar normalised distance matrix to the normalised global matrix. The cumulative proportion of genes within each SOM cluster below a certain correlation threshold was determined (Fig. 7), and the area under these curves was used to identify the three SOM clusters with the highest proportion of highly correlated genes.

### Plant research statement

Both Tapidor and Westar, as *Brassica napus* cultivars, are covered by Annex 1 of the International Treaty on Plant Genetic Resources for Food and Agriculture. The authors assert that all institutional, national, and international guidelines and legislation were followed during study. Seeds for Tapidor and Westar can be obtained by contacting Dr. Rachel Wells (rachel.wells@jic.ac.uk).

### Supplementary Information

Supplementary Figure S1.

Supplementary Figure S2.

Supplementary Figure S3.

Supplementary Figure S4.

Supplementary Figure S5.

Supplementary Figure S6.

Supplementary Figure S7.

Supplementary Table S1.

Supplementary Table S2.

Supplementary Table S3.

Supplementary Table S4.

Supplementary Table S5.

Supplementary Table S6.

Supplementary Legends.

## Data Availability

The raw reads have been deposited in the NCBI Sequence Read Archive under the BioProject numbers PRJNA398789 and PRJNA565743.
