# Supplementary figures and images for: A transcriptomic time-series reveals differing trajectories during pre-floral development in the apex and leaf in winter and spring varieties of Brassica napus
Source: Sci Rep. 2024 Feb 12;14:3538. doi: 10.1038/s41598-024-53526-x (PMC10861513; doi:10.1038/s41598-024-53526-x)

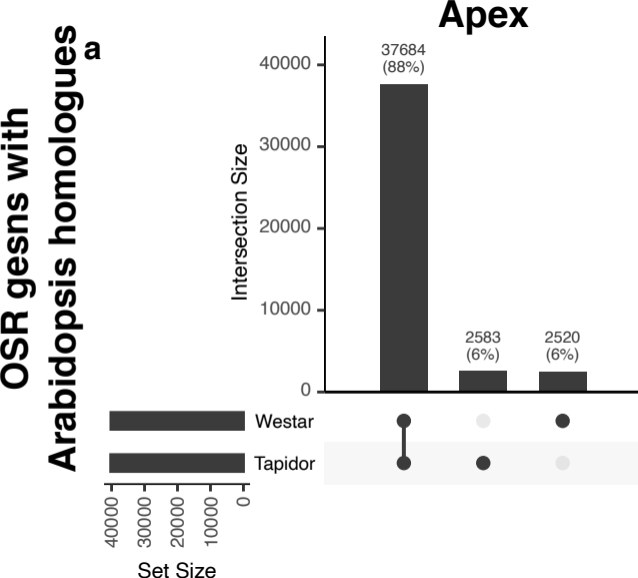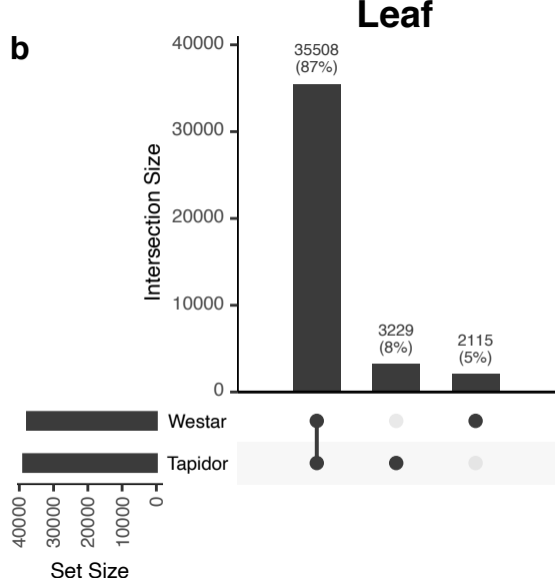

Supplement: Supplementary file 1 — Supplementary Figure S1. [file 41598_2024_53526_MOESM1_ESM.pdf]

# OSR genes with Arabidopsis homologues

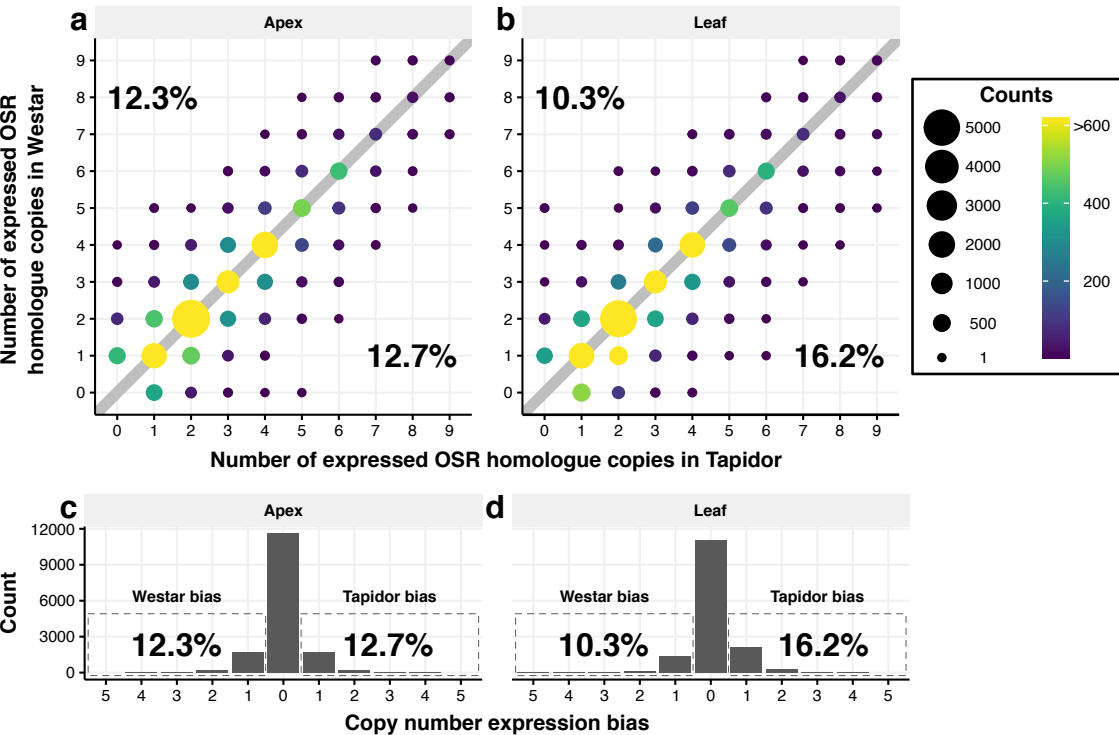

Supplement: Supplementary file 2 — Supplementary Figure S2. [file 41598_2024_53526_MOESM2_ESM.pdf]

# Genes with *A. thaliana* annotation

**a**

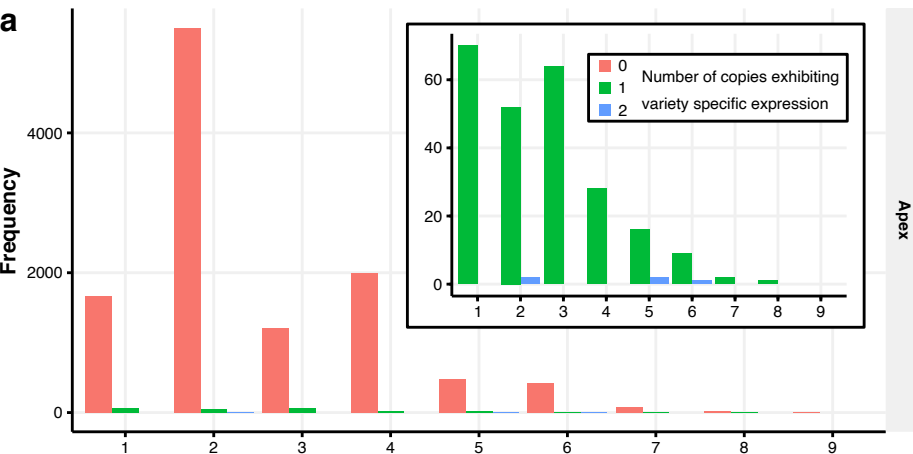

**b**

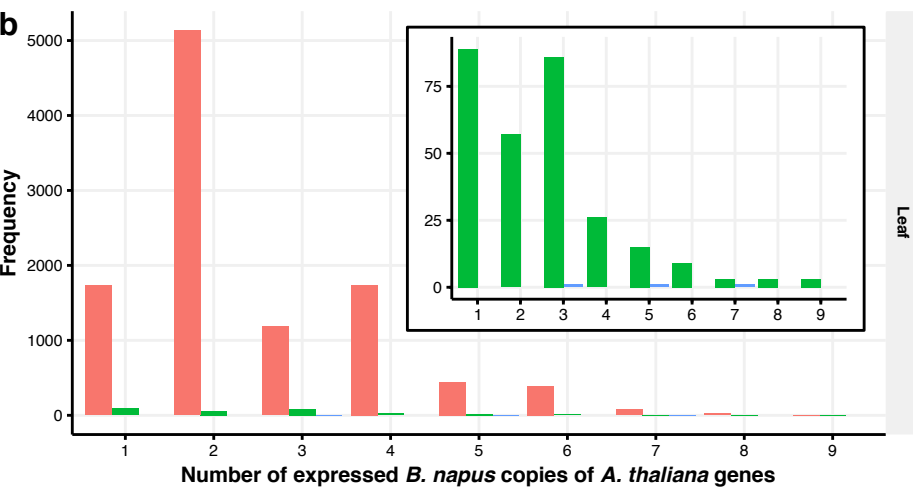

Supplement: Supplementary file 3 — Supplementary Figure S3. [file 41598_2024_53526_MOESM3_ESM.pdf]

# Genes in FLOR-ID database

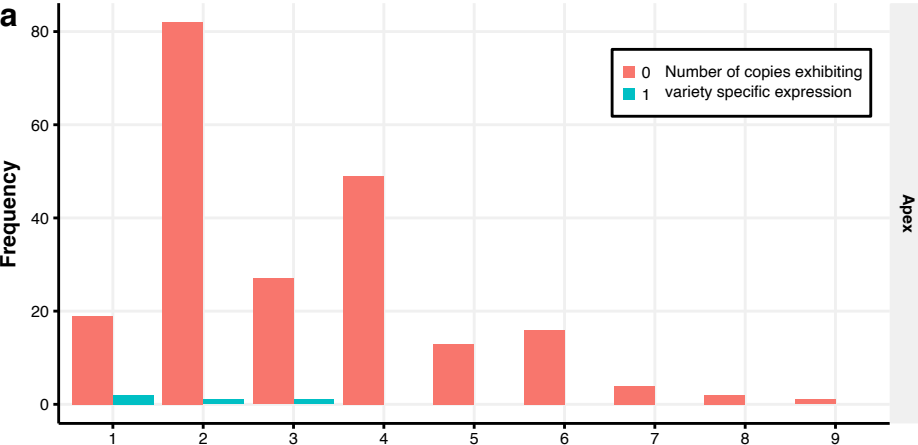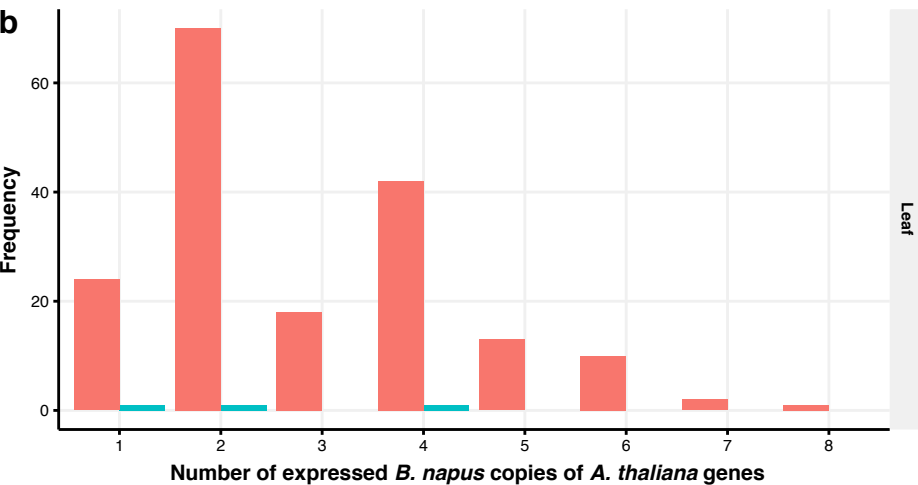

Supplement: Supplementary file 4 — Supplementary Figure S4. [file 41598_2024_53526_MOESM4_ESM.pdf]

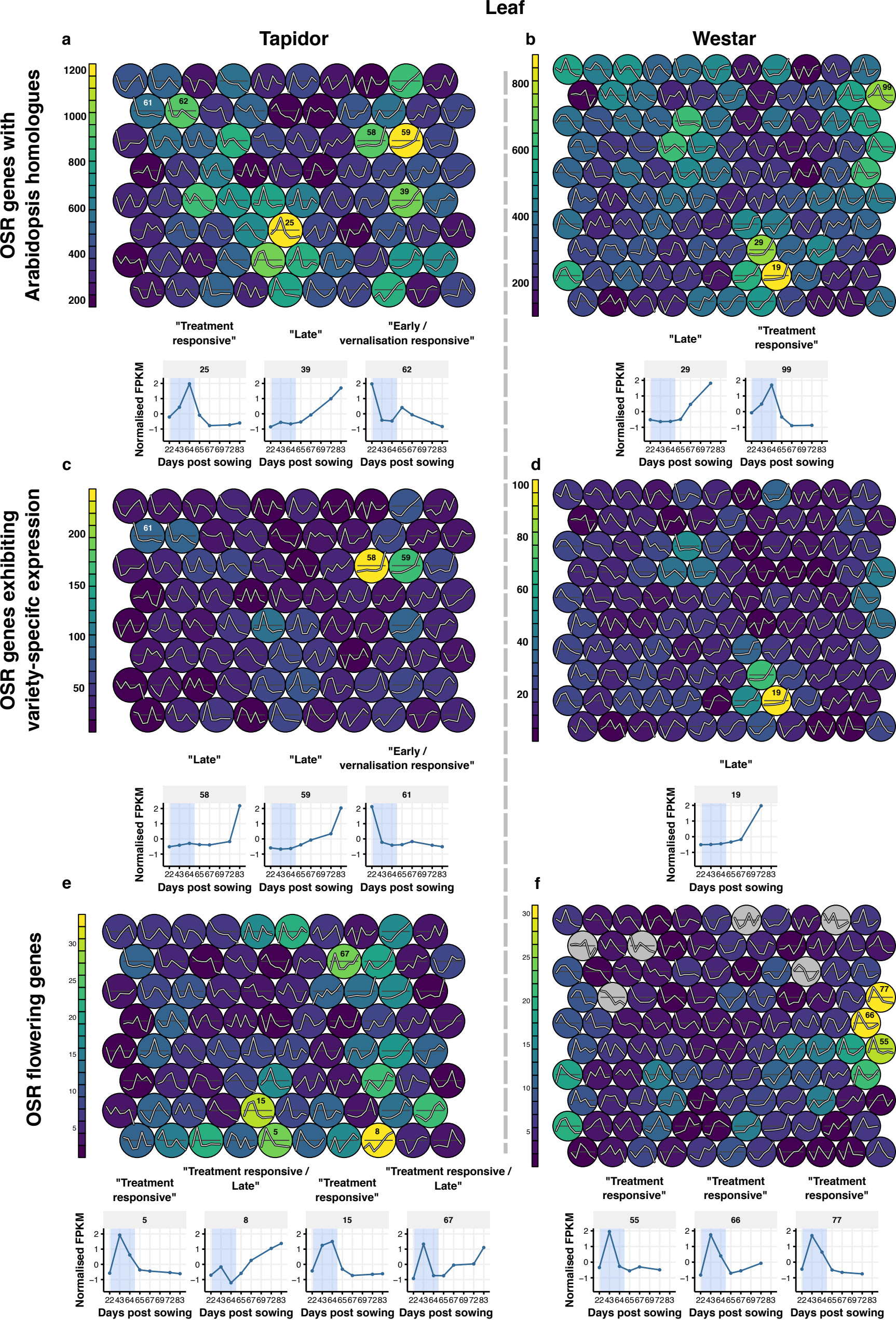

Supplement: Supplementary file 6 — Supplementary Figure S6. [file 41598_2024_53526_MOESM6_ESM.pdf]

Apex

A Genome

C Genome

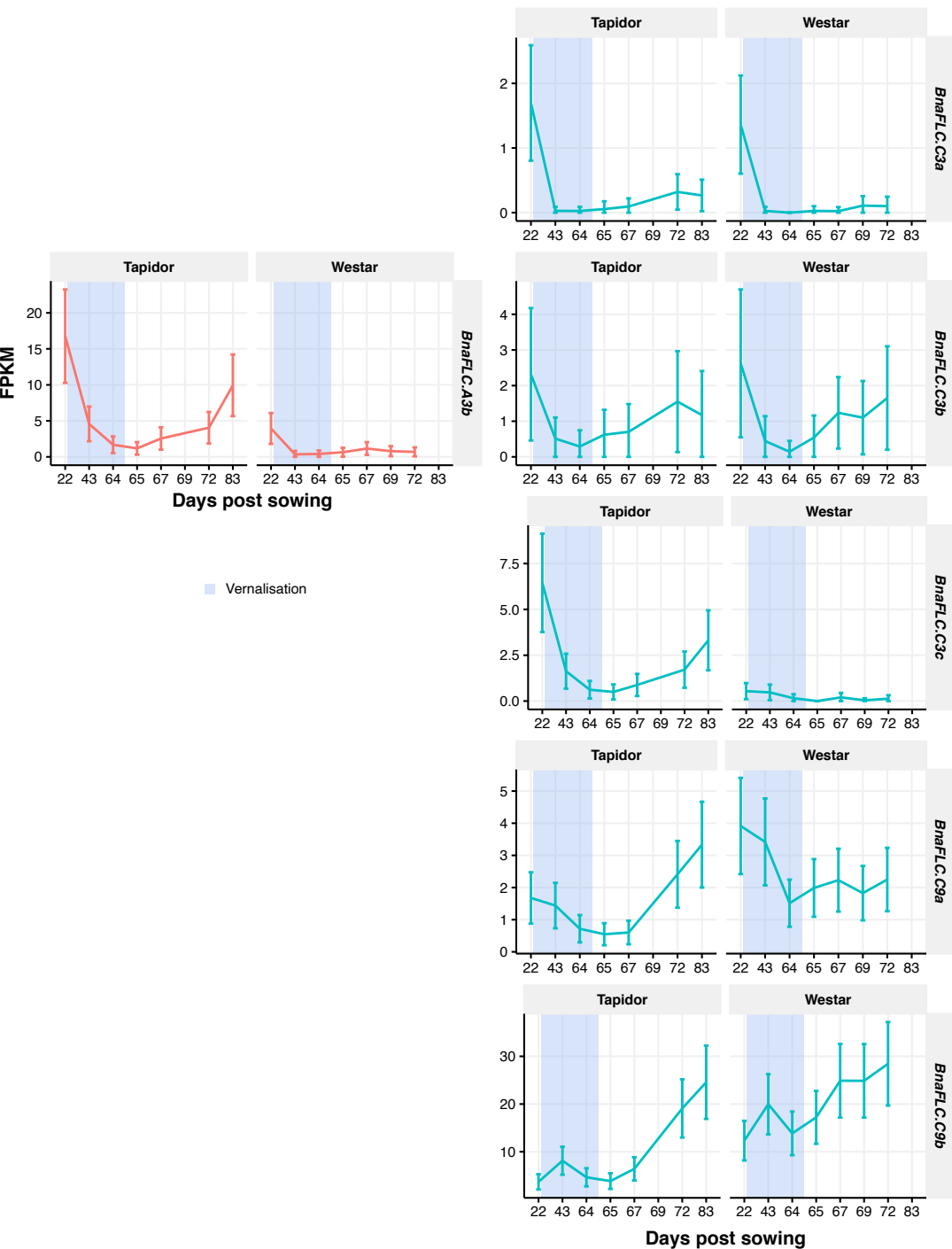

Leaf

A Genome

C Genome

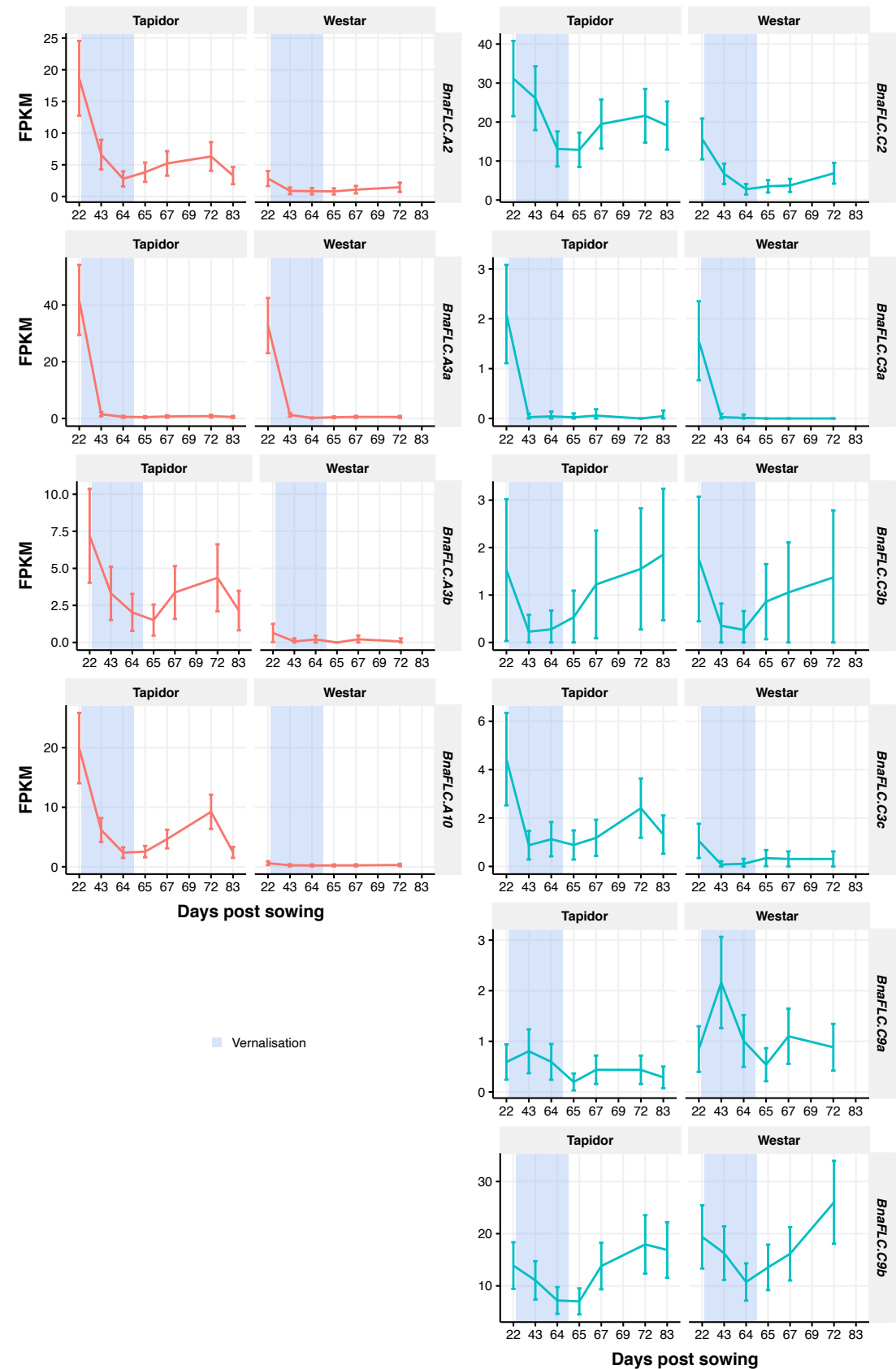

Supplement: Supplementary file 7 — Supplementary Figure S7. [file 41598_2024_53526_MOESM7_ESM.pdf]
