# Supplementary material for: A transcriptomic time-series reveals differing trajectories during pre-floral development in the apex and leaf in winter and spring varieties of Brassica napus
Source: Sci Rep. 2024 Feb 12;14:3538. doi: 10.1038/s41598-024-53526-x (PMC10861513; doi:10.1038/s41598-024-53526-x)
Supplement: Supplementary file 5 — Supplementary Figure S5. [file 41598_2024_53526_MOESM5_ESM.pdf]

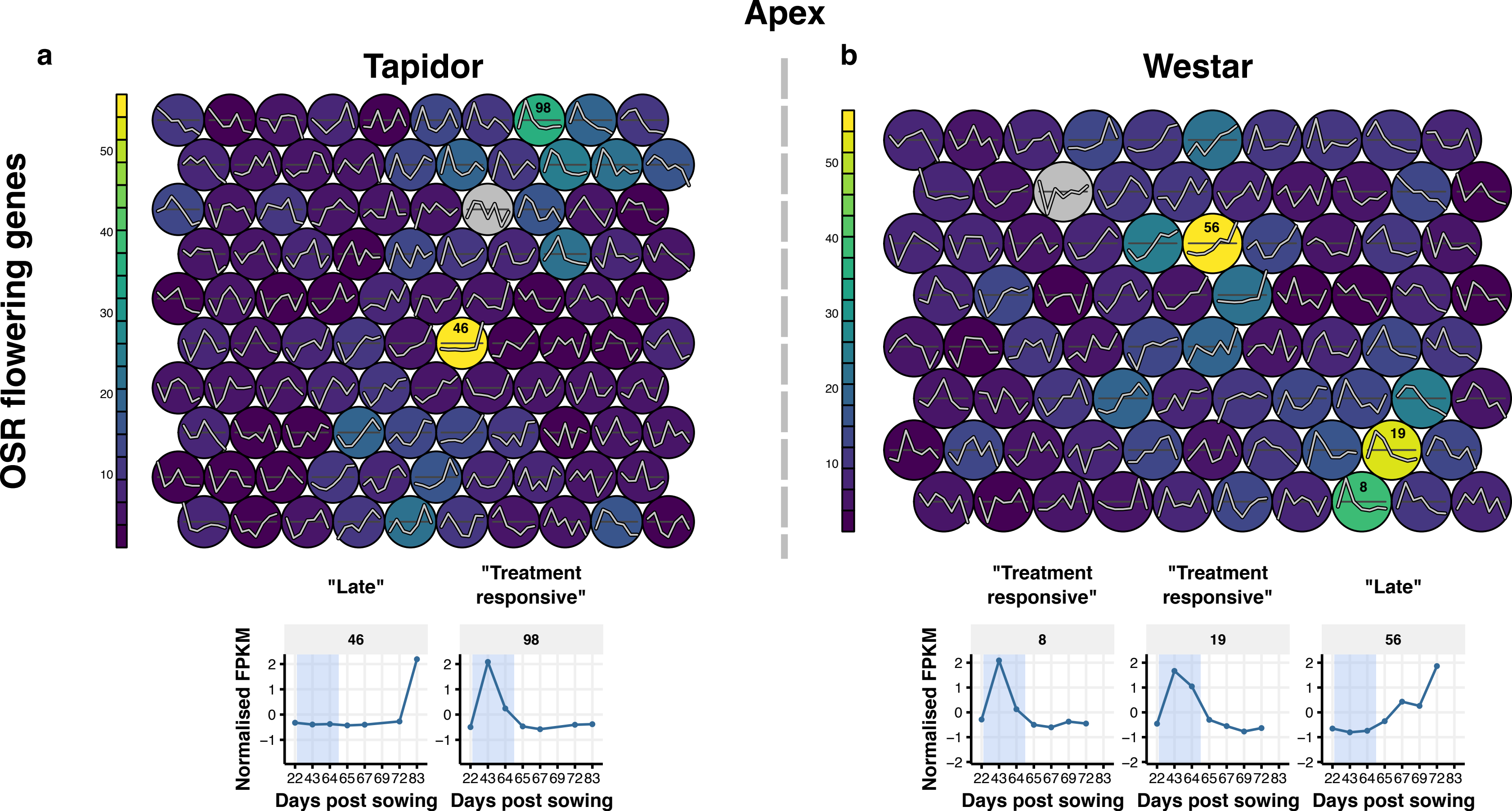

Apex
a
Tapidor
b
Westar
OSR flowering genes
98
46
56
19
8
10
20
30
40
50
"Late"
"Treatment responsive"
"Treatment responsive"
"Treatment responsive"
"Late"
Normalised FPKM
46
98
8
19
56
22 43 64 65 67 69 72 83
Days post sowing
