# Supplementary material for: A transcriptomic time-series reveals differing trajectories during pre-floral development in the apex and leaf in winter and spring varieties of Brassica napus
Source: Sci Rep. 2024 Feb 12;14:3538. doi: 10.1038/s41598-024-53526-x (PMC10861513; doi:10.1038/s41598-024-53526-x)
Supplement: Supplementary file 14 — Supplementary Legends. [file 41598_2024_53526_MOESM14_ESM.docx]

**Supplementary Figure Legends**

**Supplementary Figure 1 – There is high overlap between varieties in the sets of expressed genes**

OSR genes were regarded as expressed if their maximal expression level across the transcriptomic time series was greater than, or equal to, 2.0 FPKM. All OSR genes with Arabidopsis homologues were considered. Percentages have been rounded to the closest integer.

**Supplementary Figure 2 – The majority of Arabidopsis genes have the same number of expressed OSR orthologues in each variety**

For these plots all OSR genes with Arabidopsis homologues were considered. **a, b** OSR homologue families were defined as OSR genes which have the same Arabidopsis gene as their highest scoring BLAST hit. Expressed OSR genes were determined as those that had a maximal expression value above or equal to 2.0 FPKM at one or more time points in the time series. The size and colour of the circles indicate the number of data points at that position. The upper limit of the colour scale is the maximal off-diagonal value. Points on the diagonal, grey line represent OSR homologue families that have equal numbers of homologues expressed in both Tapidor and Westar. The left most percentage within each graph represent the percentage of Arabidopsis genes that have more homologues expressed in Westar, whereas the right most percentage is the corresponding percentage for Tapidor. **c, d** Bar charts sum the number of data points on the diagonals in **a** and **b**, with copy number expression bias determined as the absolute difference between the number of expressed genes in a OSR homologue family in both Tapidor and Westar.

**Supplementary Figure 3 – Extent of compensatory homologue expression for all OSR genes**

Only Arabidopsis genes that have the same number of homologues expressed in both Tapidor and Westar (points that lie on the diagonal grey line in Supplementary Figure 2) are considered. These are separated by those that have zero, one, or two homologues that exhibit compensatory expression behaviour. The inset displays the same data as the main figure, but without the bars corresponding to Arabidopsis genes with zero homologues that exhibit compensatory behaviour. Very few instances of compensation are observed between homologues in both the apex (**a**) and the leaf (**b**).

**Supplementary Figure 4 – Extent of compensatory homologue expression among OSR flowering genes**

Only Arabidopsis flowering time genes that have the same number of homologues expressed in both Tapidor and Westar (points that lie on the diagonal grey line in Figure 5 in the main text) are considered. These are separated by those that have zero or one homologue that exhibits compensatory expression behaviour. The inset displays the same data as the main figure, but without the bars corresponding to Arabidopsis flowering time genes with zero homologues that exhibit compensatory behaviour. Very few instances of compensation are observed between homologues in both the apex (**a**) and the leaf (**b**).

**Supplementary Figure 5 – Self-organising maps based on apex transcriptome with floral genes mapped to it**

As for Figure 5 in the main text, showing the mapping of OSR genes that show sequence similarity to Arabidopsis genes in the FLOR-ID database of floral genes^48^ to the SOM.

**Supplementary Figure 6 – Self-organising maps based on leaf transcriptomes reveal that variety-specific genes tend to be expressed at the end of the time series**

As for Figure 5 in the main text and Supplementary Figure 5, for the leaf transcriptome.

**Supplementary Figure 7 – All other *BnaFLC* copies not shown in Figure 8**

Expression traces across the developmental time series in apex and leaf tissues for *BnaFLC* genes not shown in Figure 8 in the main text. The blue segments indicate the time points sampled during the vernalisation treatment.

**Supplementary Table legends**

**Supplementary Table 1 – Results of gene ontology term enrichment for variety-specific genes in the apex**

Enriched biological process (“_bp” suffix) and molecular function (“_mf” suffix) gene ontology terms for variety-specific genes in the apex in both varieties.

**Supplementary Table 2 – Results of gene ontology term enrichment for variety-specific genes in the leaf**

Enriched biological process (“_bp” suffix) and molecular function (“_mf” suffix) gene ontology terms for variety-specific genes in the leaf in both varieties.

**Supplementary Table 3 – Results of testing self-organising map clusters for enrichment of genes expressed in a variety-specific manner**

Fisher test results to determine enrichment of variety-specific genes in each self-organising map cluster for both tissues and both varieties.

**Supplementary Table 4 – List of genes expressed in each variety and tissue and the self-organising map clusters to which they are mapped**

The self-organising map clusters to which expressed OSR genes are mapped. BLAST homology mapping information, as well as whether that gene is expressed in a variety-specific manner or is annotated as a flowering time gene, is also supplied in the table.

**Supplementary Table 5 – Table of gene model mappings for the OSR genes mentioned in this study**

Contains BLAST homology mapping information to both the Arabidopsis and the *Brassica napus* pantranscriptome ^81^. Notably, the adjacent *BnaFLC.C3b* and *BnaFLC.C3c* are mapped to the same pantranscriptome gene model, suggesting that they are a split gene model.

**Supplementary Table 6 – List of gene ontology terms enriched in every self-organising map cluster**

Biological process gene ontology terms enriched in every self-organising map cluster.
